# Arbuscular Mycorrhizal Fungi Regulate Polyamine Homeostasis in Roots of Trifoliate Orange for Improved Adaptation to Soil Moisture Deficit Stress

**DOI:** 10.3389/fpls.2020.600792

**Published:** 2021-01-12

**Authors:** Ying-Ning Zou, Fei Zhang, Anoop K. Srivastava, Qiang-Sheng Wu, Kamil Kuča

**Affiliations:** ^1^College of Horticulture and Gardening, Yangtze University, Jingzhou, China; ^2^ICAR-Central Citrus Research Institute, Nagpur, India; ^3^Department of Chemistry, Faculty of Science, University of Hradec Kralove, Hradec Kralove, Czechia

**Keywords:** citrus, mycorrhiza, polyamine, *Poncirus trifoliata*, water deficit

## Abstract

Soil arbuscular mycorrhizal fungi (AMF) enhance the tolerance of plants against soil moisture deficit stress (SMDS), but the underlying mechanisms are still not fully understood. Polyamines (PAs) as low-molecular-weight, aliphatic polycations have strong roles in abiotic stress tolerance of plants. We aimed to investigate the effect of AMF (*Funneliformis mosseae*) inoculation on PAs, PA precursors, activities of PA synthases and degrading enzymes, and concentration of reactive oxygen species in the roots of trifoliate orange (*Poncirus trifoliata*) subjected to 15 days of SMDS. Leaf water potential and total chlorophyll levels were comparatively higher in AMF-inoculated than in non-AMF-treated plants exposed to SMDS. Mycorrhizal plants recorded a significantly higher concentration of precursors of PA synthesis such as L-ornithine, agmatine, and *S*-adenosyl methionine, besides higher putrescine and cadaverine and lower spermidine during the 15 days of SMDS. AMF colonization raised the PA synthase (arginine decarboxylase, ornithine decarboxylase, spermidine synthase, and spermine synthase) activities and PA-degrading enzymes (copper-containing diamine oxidase and FAD-containing polyamine oxidase) in response to SMDS. However, mycorrhizal plants showed a relatively lower degree of membrane lipid peroxidation, superoxide anion free radical, and hydrogen peroxide than non-mycorrhizal plants, whereas the difference between them increased linearly up to 15 days of SMDS. Our study concluded that AMF regulated PA homeostasis in roots of trifoliate orange to tolerate SMDS.

## Introduction

Polyamines (PAs) are low-molecular-weight, aliphatic polycationic compounds that widely occur in prokaryotic and eukaryotic cells (Takahashi, 2020). In plants, the abundant PAs include putrescine (Put), spermidine (Spd), and spermine (Spm) as well as the less abundant cadaverine (Cad) (Upadhyay et al., 2020). Plant Put originates from L-arginine (L-Arg) and L-ornithine (L-Orn) via the catalytic action of arginine decarboxylase (ADC) and ornithine decarboxylase (ODC), respectively (Liu et al., 2015). Put serves as the substrate for the Spd biosynthesis under the combination with decarboxylated *S*-adenosyl methionine (SAM) and then Spm biosynthesis via the action of *S*-adenosyl methionine decarboxylase (SAMDC), spermidine synthases (SPDS), and spermine synthases (SPMS) (Salloum et al., 2018). PA catabolism in plants is operated through copper-containing diamine oxidase (CuAO) and FAD-containing polyamine oxidase (PAO) predominantly localized in the cell wall (Šebela et al., 2001). CuAO mainly catalyzes the oxidation of Put and Cad, and PAO catalyzes the oxidation of Spd and Spm. Hydrogen peroxide (H_2_O_2_), a kind of reactive oxygen species (ROS), is the byproduct of PA catabolism.

A large number of studies have shown that plant PAs are involved in a variety of responses against abiotic stress (Alcázar et al., 2020; Upadhyay et al., 2020; Zhang et al., 2020). The roles of PAs in response to plant stress include (1) acting as a compatible solute along with γ-aminobutyric acid, glycinebetaine, and proline; (2) stabilizing cellular organellar membranes and macromolecules; (3) acting as scavengers of ROS and triggering the antioxidant defense system; (4) considering as signal molecules in the ABA-regulated stress responses; (5) regulating the ion channels; and (6) participating in programmed cell death (Gupta et al., 2013; Minocha et al., 2014). These multiple roles of PAs suggest important cellular functions in plants while coping with abiotic stress.

Soil moisture deficit stress (SMDS) is one of the abiotic stresses, which seriously affects the growth and yield of crops (Muthusamy et al., 2014). It is well established that arbuscular mycorrhizal fungi (AMF) in soil colonize host roots, thereby establishing the arbuscular mycorrhizal (AM) symbionts. Such symbionts enhance the drought tolerance of host plants through a series of morphological, physiological, and molecular mechanisms (Baslam and Giocoechea, 2012; Wu et al., 2013, 2019; Millar and Bennett, 2016; Huang et al., 2017; Venice et al., 2017; He et al., 2019, 2020; Lokhandwala and Hoeksema, 2019; Zou et al., 2019, 2020; Cheng et al., 2020). In maize, AMF triggered Put catabolism into γ-aminobutyric acid along with an improved N-assimilation, which is an important metabolic process in mycorrhizal plants in response to drought stress (Hu and Chen, 2020). A higher Put and Cad and lower Spd and Spm level in mycorrhizal trifoliate orange (*Poncirus trifoliata* L. Raf.) compared with non-mycorrhizal plants is reported under drought stress (Zhang et al., 2020). Proteomics techniques further showed that when the mycelium of ericoid mycorrhizal fungi was exposed to excess of zinc and cadmium, agmatinase (an enzyme associated with PA biosynthesis) was accumulated in mycorrhizal plants in response to abiotic stress (Chiapello et al., 2015). However, Luo (2009) observed comparatively lower Put levels in mycorrhizal over non-mycorrhizal trifoliate orange seedlings exposed to drought stress. In alfalfa plants, AMF inoculation induced an increase in concentration of Spd and Spm under drought stress (Goicoechea et al., 1998). These inconsistent responses on PA changes indicate that changes of PA pool are one of the mechanisms responsible for improved adaptation under abiotic stress. The relationship between AMF and PAs of host plants is rather complex, especially with regard to changes in the PA homeostasis and metabolic pathways involved.

Citrus is globally one of the most traded fruit crops (Srivastava and Singh, 2008). Likewise, trifoliate orange is the most extensively used rootstock for satsuma mandarin (*Citrus unshiu* Marc.) grown on Oxisols, Alfisols, and Ultisols, where exposure to SMDS is a common feature (Srivastava and Singh, 2009). Trifoliate orange is a drought-sensitive rootstock. In a plant, the roots act as first sensor to perceive the soil water deficit (Davies et al., 1994). Considering AMF as root colonizer, biochemical parameters in roots are expected to be more revealing than analysis of any other plant parts. In this study, we hypothesized that AMF inoculation could modulate root PA homeostasis of host plants to cope with drought stress. In this background, we studied the changes in four PA levels, four PA precursor levels, and PA synthase and catabolase activities, accompanied with ROS and degree of membrane lipid peroxidation in roots of trifoliate orange inoculated with AMF followed by exposure to SMDS under controlled-environment conditions.

## Materials and Methods

### Preparation of Mycorrhizal Fungal Inoculum

An arbuscular mycorrhizal fungus, *Funneliformis mosseae* (Nicol. & Gerd.) C. Walker & A. Schüßler, was chosen based on its ability in enhancing drought tolerance of trifoliate orange on inoculation with this strain (He et al., 2020). The fungal strain was propagated with identified spores and white clover in pots for 3 months. At harvest, the aboveground part of white clover was removed, and both roots and growth substrates were collected as the mycorrhizal fungus inoculant, which contained soil hyphae, AMF-colonized root segments, and spores (16 spores g^–1^).

### Plant Culture

The seeds of trifoliate orange were surface sterilized with 75% of ethyl alcohol solutions for 10 min, rinsed with distilled water, and placed in autoclaved sand for germination under 26°C and 72% relative air humidity. A month later, the seedlings of four-leaf age and uniform in size were transplanted into 2.3-L plastic pots filled with 2.8 kg of autoclaved mixture of soil and sand (1:1, *v*/*v*). The soil belonged to the Ferralsol (FAO system) with pH 6.2, Brays-P 17.43 mg kg^–1^, and soil organic carbon content 10.68 mg kg^–1^. At the same time, 100 g of mycorrhizal inoculum was added into the rhizosphere of potted seedlings in each pot. An equal amount of sterilized mycorrhizal inoculant, plus 2 ml inoculum filtrate using 25-μm filters, was applied into the seedlings’ rhizosphere as the non-AMF treatment. The non-AMF- and AMF-inoculated seedlings were kept at 75% of maximum field water capacity (24.11%), corresponding to well-watered (WW) status, in a growth chamber of Yangtze University (Jingzhou, China) maintained at 900 μmol m^–2^ s^–1^ photon flux density, 28/20°C day/night temperature, and 68% relative air humidity. During this experiment, the plants did not receive any nutrient solution.

Fifteen weeks later, soil moisture in all the treated pots was adjusted to soil WW status (18.08%), designed as 0 days, before SMDS. Subsequently, SMDS priming was executed by stopping the water supply. AMF- and non-AMF-inoculated plants were harvested at 0th, 5th, 10th, and 15th day of SMDS. During the 15 days of SMDS, the soil moisture content reduced from 18.08 to 4.18% in AM plants and from 18.08 to 4.24% in non-AM plants. At each harvest, 12 seedlings (three seedlings per pot) of four pots of each treatment were harvested. As a result, the experiment was laid out in a completely randomized blocked design with AMF and non-AMF inoculations under SMDS of 0, 5, 10, and 15 days. A total of 32 pots containing 16 AMF-inoculated pots and 16 non-AMF-inoculated pots were used. The experiment was conducted for 120 days.

### Determination of Plant Growth and Root Mycorrhizal Colonization

Plant growth parameters such as plant height, stem diameter, and leaf number per plant were recorded before SMDS began. At 0 days of SMDS, AMF- and non-AMF-treated plants were harvested and divided into shoots and roots, followed by determination of their biomass. And then, at each harvest, the treated plants were divided into shoots and roots. Part root samples were frozen with liquid nitrogen and then stored at −80°C for the subsequent analysis of biochemical parameters. Six 1-cm-long root segments per plant were cleared in 10% KOH solution and stained by trypan blue in lactic acid (Phillips and Hayman, 1970). Root mycorrhizas were observed under an optical microscope, and the rate of root mycorrhizal colonization (%) was calculated as the percentage of AMF-colonized root lengths versus total observed root lengths (Zhang et al., 2018).

### Determination of Leaf Water Potential and Total Chlorophyll Contents

Total chlorophyll (*a* + *b*) content in leaves was determined using the procedure as suggested by Lichtenthaler and Wellburn (1983) by extraction with 80% acetone. Leaf water potential (*Ψ*) was measured by PSΨPRO matched with a dew point microvoltmeter (HR-33T; Wescor, Logan, UT, United States) on a fully expanded second leaf at the top.

### Determination of Root PA Contents

Root PAs (Spm, Spd, Put, and Cad) and precursors (L-Arg, L-Orn, agmatine, and SAM) to PA synthesis were extracted and quantified according to the protocol outlined by Ducros et al. (2009) with minor modification. A 50-mg fresh root sample was ground in an ice bath with 0.5 ml of pre-cooled solutions containing acetonitrile, methanol, and distilled water (2:2:1, *v*/*v*), followed by ultrasonic treatment for 5 min. The samples were placed at −20°C for 1 h and then centrifuged at 12,000 × *g* for 15 min. The 0.1-ml supernatant was mixed with 50 μl sodium carbonate solutions and 50 μl 20 mg/ml dansulfonyl chloride solution at 40°C for 1 h in the dark. The mixture was incubated with 1% formic acid for 30 s and centrifuged at 12,000 × *g* for 15 min. A total of 80 μl supernatants were used for analysis of free PAs and precursors by the ultra-high performance liquid chromatograph (UHPLC)–MS. The Agilent 1290 Infinity II series (Agilent Technologies, Santa Clara, CA, United States) UHPLC was used, and the liquid chromatography column namely ACQUITY UPLC HSS T3 (100 × 2.1 mm, 1.8 μm; Waters) was used for the separation. The A phase of the liquid chromatograph was 10 mM formic acid solution, while the B phase was acetonitrile solution using the column temperature of 35 ± 4°C as the sample tray temperature and 1 μl as the sample volume. Mass spectrometry data acquisition and quantitative analysis of target compounds were carried out through Agilent MassHunter Work Station Software (B.08.00; Agilent Technologies).

### Determination of Root PA-Metabolized Enzyme Activities

Root samples were washed in precooled phosphate buffer (0.01 M, pH 7.4), followed by incubation of 0.1 g treated root samples with 0.9 ml 0.01 M (pH 7.4) phosphate buffer. The homogenate was placed in an ice bath for ultrasonic crushing (20 kHz) with a total of three crushings, every crushing lasting for 5 s intermittently involving of the interval of 3 s. Finally, the homogenate was centrifuged at 5,000 × *g* for 10 min, and the supernatant was taken for the analysis of PA-metabolized enzyme activities, based on ELISA, sourced from Shanghai Enzyme-Linked Biotechnology Co. Ltd (Shanghai, China). All the determinations were carried out according to the recommended user’s manuals. The following kits in the order of ml090024-S, ml090861-S, ml073540-S, ml187041-S, ml067255-S, and ml096680-S, respectively, were used for the analysis of PAO, CuAO, ADC, ODC, SPMS, and SPDS.

### Determination of ROS and Degree of Membrane Lipid Peroxidation

The H_2_O_2_ in roots was extracted with 0.1% trichloroacetic acid solution and determined as per procedure put forward by Velikova et al. (2000). Root superoxide anion free radical (O_2_^–^) content was assayed using the procedure described by He et al. (2020). Root malonaldehyde (MDA, the degree of membrane lipid peroxidation) concentration was determined according to Sudhakar et al. (2001).

### Statistical Analysis

The measurement of all variables was replicated four times. Experimental data (mean ± SD, *n* = 4) generated were performed by ANOVA according to SAS (SAS Institute, Cary, NC, United States). The percentage regarding root AMF colonization rate required arcsine transformation before ANOVA. Duncan’s multiple range tests were used to compare the significant differences between treatments at the 5% level.

## Results

### Changes in Plant Growth

Compared with non-AMF-inoculated seedlings, AMF-inoculated seedlings recorded significantly (*P* < 0.05) higher plant height, stem diameter, leaf number per plant, shoot biomass, and root biomass by 68%, 16%, 44%, 69%, and 46%, respectively, before SMDS began (Table 1).

**TABLE 1:** Plant growth of trifoliate orange seedlings inoculated with *Funneliformis mosseae* before soil moisture deficit stress (SMDS).

Treatments	Plant height (cm)	Stem diameter (cm)	Lear number per plant	Shoot biomass (g FW/plant)	Root biomass (g FW/plant)
−AMF	15.30 ± 1.3b	0.31 ± 0.04b	16 ± 1b	1.02 ± 0.14b	1.62 ± 0.24b
+AMF	25.7 ± 4.4a	0.36 ± 0.04a	23 ± 3a	1.72 ± 0.30a	2.37 ± 0.32a

*Data (mean ± SD, *n* = 4) followed by different letters in the same column indicate significant (*P* < 0.05) differences.*

### Changes in Root Mycorrhizal Colonization

**FIGURE 1:**
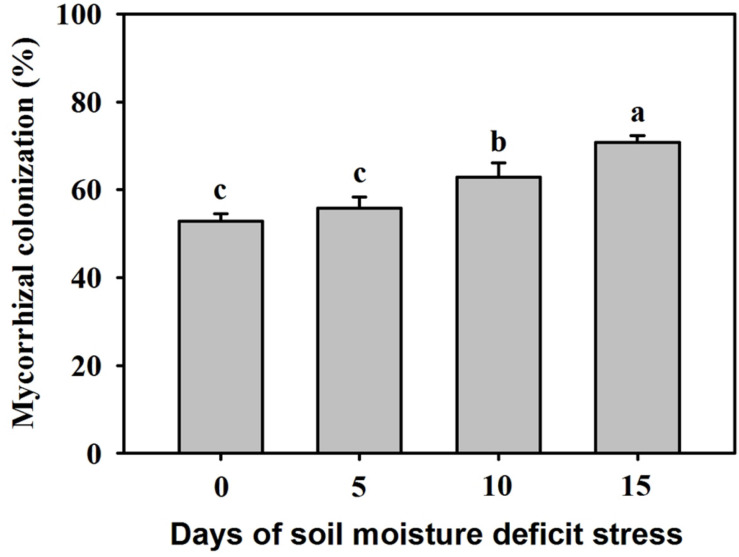
Root mycorrhizal colonization of trifoliate orange seedlings inoculated with *Funneliformis mosseae* during 0-15 days of soil moisture deficit stress (SMDS). Data (mean ± SD, *n* = 4) followed by different letters above the bars indicate significant (*P* < 0.05) differences.

Mycorrhizal colonization is an indicator of a given mycorrhizal species to colonize the roots of host plants. In this study, no mycorrhizal structure was observed in the roots of non-AMF-inoculated plants. The root mycorrhizal colonization ranged from 52.81 to 70.74%, and the soil water deficit markedly affected the root mycorrhizal colonization (Figure 1). Compared with the inoculated plants at 0 SMDS, root AMF colonization showed no significant difference versus inoculated plants at 5 days of SMDS. Interestingly, root AMF colonization was 19% and 34% significantly (*P* < 0.05) higher in the inoculated seedlings exposed to 10 days and 15 days of SMDS than exposed to 0 days, respectively.

### Changes in Leaf *Ψ*

**FIGURE 2:**
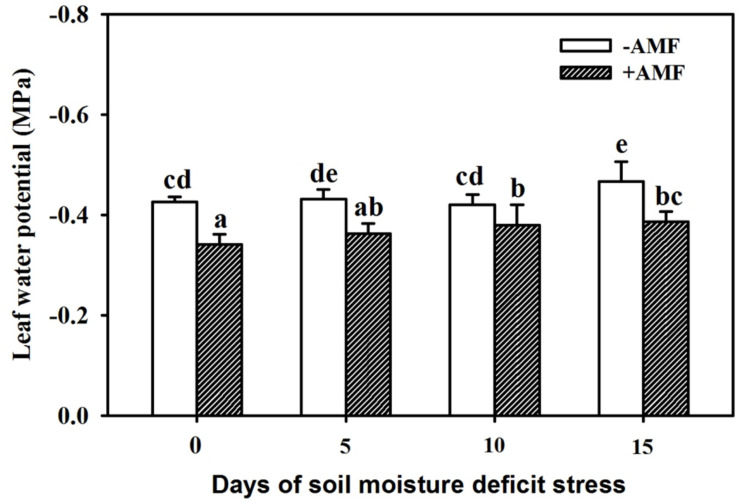
Leaf water potential of trifoliate orange seedlings inoculated with and without *Funneliformis mosseae* during exposure to soil moisture deficit stress (SMDS) of 0-15 days. Data (mean ± SD, *n* = 4) followed by different letters above the bars indicate significant (*P* < 0.05) differences.

With an increase in duration of SMDS, leaf *Ψ* of plants showed a decreasing trend (Figure 2). SMDS up to 5 days showed no significant effect on leaf *Ψ* in AMF- and non-AMF-inoculated plants, and thereafter up to 15 days of SMDS *Ψ* consistently decreased as compared with SMDS for 0 days. The AMF-treated plants recorded 20%, 16%, 9%, and 17% significantly (*P* < 0.05) higher leaf *Ψ* than non-AMF-treated plants at 0, 5, 10, and 15 days of SMDS, respectively.

### Changes in Leaf Total Chlorophyll Concentration

**FIGURE 3:**
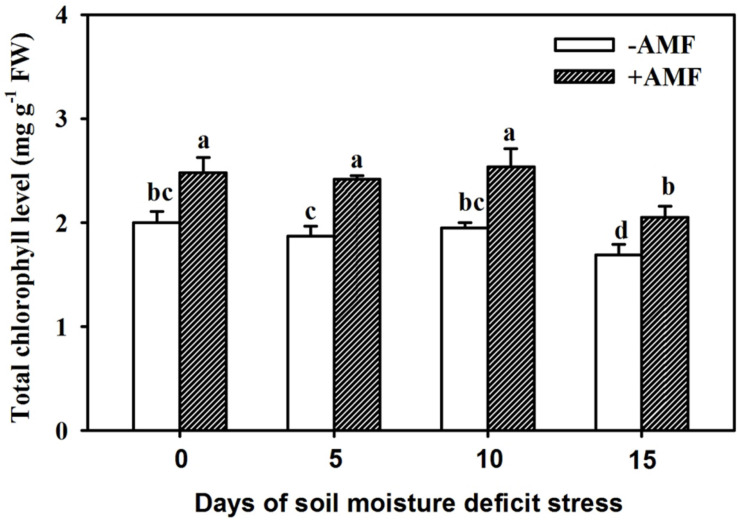
Leaf total chlorophyll levels of trifoliate orange seedlings inoculated with and without *Funneliformis mosseae* during exposure to soil moisture deficit stress (SMDS) of 0-15 days. Data (mean ± SD, *n* = 4) followed by different letters above the bars indicate significant (*P* < 0.05) differences.

The process of drought stress in soil affected leaf total chlorophyll content, to some extent, and the 15-day drought stress strongly inhibited total leaf chlorophyll contents (Figure 3). Inoculation with AMF produced a significant (*P* < 0.05) increase in total chlorophyll content by 24%, 29%, 30%, and 21%, respectively, at 0, 5, 10, and 15 days of SMDS compared with non-AMF treatment.

### Changes in Concentration of Precursors of PAs in Roots

**FIGURE 4:**
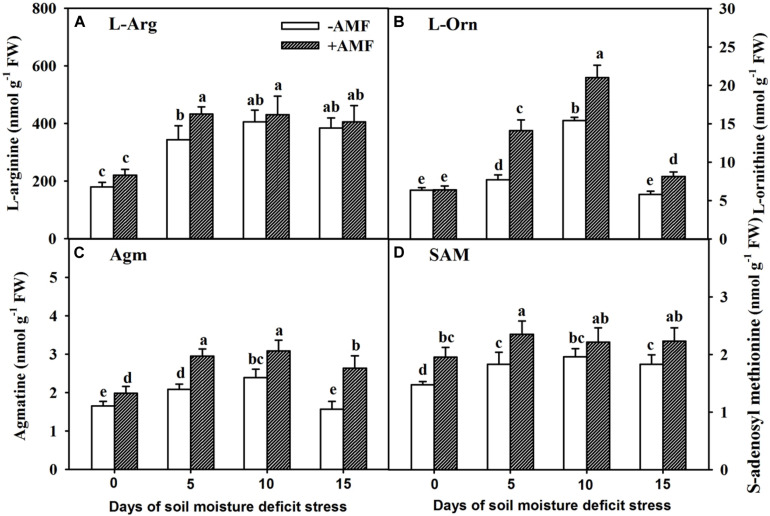
Concentrations of root L-arginine (L-Arg) **(A)**, L-ornithine (L-Orn) **(B)**, agmatine (Agm) **(C)**, and *S*-adenosyl methionine (SAM) **(D)** of trifoliate orange seedlings inoculated with and without *Funneliformis mosseae* during exposure to soil moisture deficit stress (SMDS) of 0-15 days. Data (mean ± SD, *n* = 4) followed by different letters above the bars indicate significant (*P* < 0.05) differences.

In the course of 15 days of SMDS, concentration of different precursors (L-Arg, L-Orn, Agm, and SAM) for the synthesis of PAs in roots showed an initial trend of increase followed by decreasing trend later (Figure 4). AMF inoculation did not change root L-Arg concentrations at 0, 10, and 15 days of SMDS, but increased root L-Arg concentration at 5 days of SMDS by 26% over non-AMF inoculation (Figure 4A). On the other hand, root L-Orn concentration remained unchanged between AMF- and non-AMF-inoculated plants at 0 days of SMDS, and was significantly (*P* < 0.05) higher in AMF-inoculated plants than in non-AMF-inoculated plants at 5, 10, and 15 days of SMDS by 83%, 36%, and 40%, respectively (Figure 4B). AMF-inoculated plants showed 21%, 42%, 29%, and 68% significantly (*P* < 0.05) higher root Agm concentration than non-AMF-inoculated plants at 0, 5, 10, and 15 days of SMDS, respectively (Figure 4C). Similarly, AMF-inoculated plants recorded 33%, 28%, and 22% significantly (*P* < 0.05) higher root SAM concentration over non-AMF-inoculated plants at 0, 5, and 15 days of SMDS, respectively (Figure 4D).

### Changes in Root PA Concentration

**FIGURE 5:**
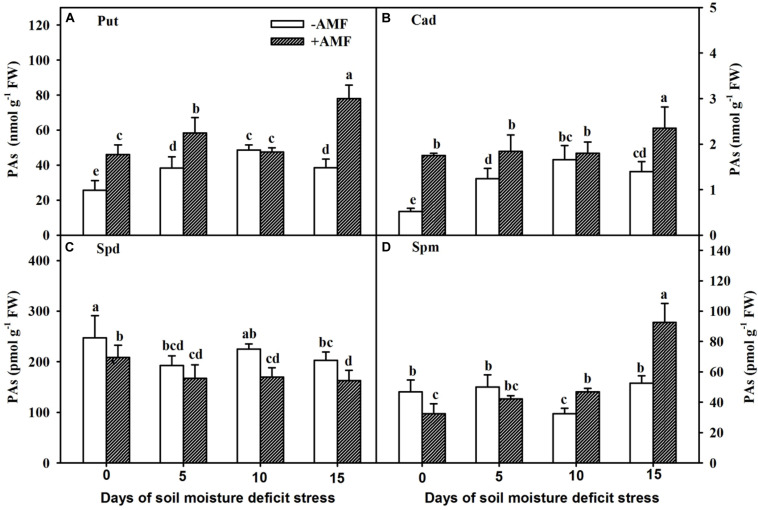
Concentrations of root putrescine (Put) **(A)**, cadaverine (Cad) **(B)**, spermidine (Spd) **(C)**, and spermine (Spm) **(D)** of trifoliate orange seedlings inoculated with and without *Funneliformis mosseae* during exposure to soil moisture deficit stress (SMDS) of 0-15 days. Data (mean ± SD, *n* = 4) followed by different letters above the bars indicate significant (*P* < 0.05) differences.

The Put levels in roots of non-AMF-inoculated plants increased gradually with the prolongation of the SMDS, reached maximum at 10 days, and then followed a declining trend (Figure 5A), while the Put levels in roots of AMF-inoculated plants reached maximum at 15 days of SMDS. Compared with non-AMF-inoculated plants, AMF-inoculated trifoliate orange seedlings showed 78%, 52%, and 103% significantly (*P* < 0.05) higher root Put level at 0, 5, and 15 days, respectively.

In non-AMF-inoculated plants, root Cad levels increased gradually with the SMDS and reached the peak at 10 days, followed by a reduction (Figure 5B). However, Cad concentration in roots of AMF-inoculated plants did not change between 0 and 10 days, and increased at 15 days. AMF inoculation significantly (*P* < 0.05) elevated the concentration of root Cad by 238%, 48%, and 68% at 0, 5, and 15 days of SMDS, respectively, compared with non-AMF treatment.

SMDS, to some degree, reduced the root Spd content but partly increased root Spm level (Figures 5C,D). AMF-inoculated plants recorded 16%, 24%, and 20% significantly (*P* < 0.05) lower root Spd content than non-AMF-inoculated plants at 0, 10, and 15 days, respectively (Figure 5C). AMF treatment significantly (*P* < 0.05) increased root Spm level by 44% and 76% at 10 and 15 days of SMDS, respectively, though reduced root Spm concentration by 30% at 0 days (Figure 5D).

### Changes in PA Synthetases in Roots

**FIGURE 6:**
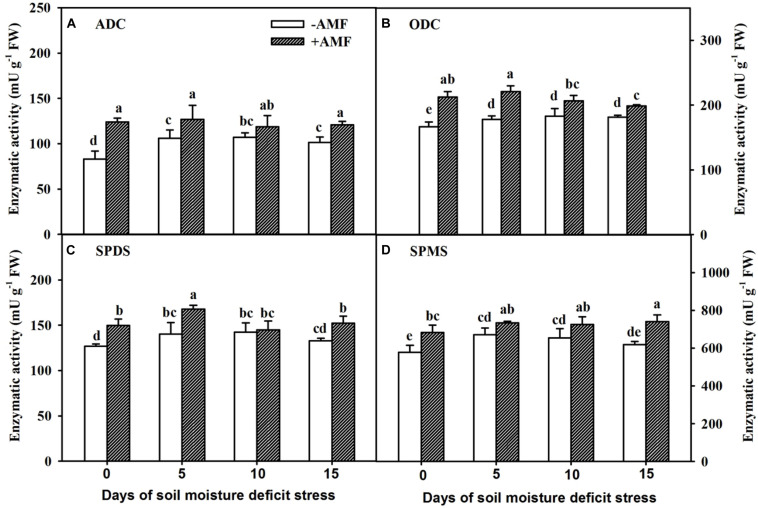
Activities of root arginine decarboxylase (ADC) **(A)**, ornithine decarboxylase (ODC) **(B)**, spermidine synthases (SPDS) **(C)**, and spermine synthases (SPMS) **(D)** of trifoliate orange seedlings inoculated with and without *Funneliformis mosseae* during exposure to soil moisture deficit stress (SMDS) of 0-15 days. Data (mean ± SD, *n* = 4) followed by different letters above the bars indicate significant (*P* < 0.05) differences.

A short 5-day SMDS induced an increase in root PA synthetase activities in non-AMF-inoculated plants and subsequently remained relatively stable (Figure 6). In AMF-inoculated plants, root ADC activity remained stable during SMDS, root SPMS activity increased slowly, root ODC activity slightly increased and then gradually decreased, and root SPDS activity showed diverse changes. AMF-inoculated plants showed significantly (*P* < 0.05) higher root ADC (Figure 6A) and SPDS (Figure 6C) activities than non-AMF-inoculated plants at 0, 5, and 15 days of SMDS, respectively. On the other hand, AMF colonization significantly (*P* < 0.05) elevated root ODC (Figure 6B) and SPMS (Figure 6D) activities at 0, 5, 10, and 15 days of SMDS, respectively, compared with non-AMF inoculation.

### Changes in PA-Degradated Enzymes in Roots

**FIGURE 7:**
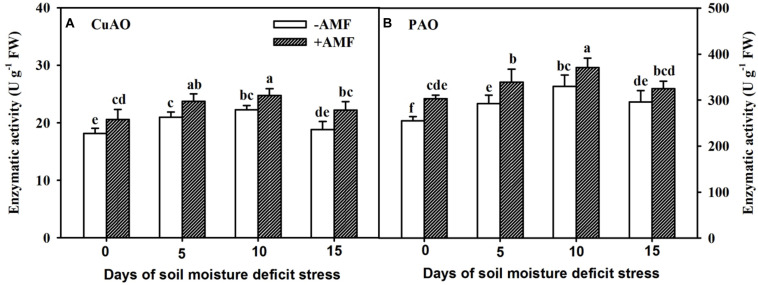
Activities of root copper-containing diamine oxidase (CuAO) **(A)** and FAD-containing polyamine oxidase (PAO) **(B)** of trifoliate orange seedlings inoculated with and without *Funneliformis mosseae* during exposure to soil moisture deficit stress (SMDS) of 0-15 days. Data (mean ± SD, *n* = 4) followed by different letters above the bars indicate significant (*P* < 0.05) differences.

Root CuAO and PAO activities in AMF- and non-AMF-inoculated trifoliate orange seedlings initially increased and then reduced, with the increasing duration of SMDS, and the highest peak was observed at 10 days (Figure 7). In the course of soil drought, AMF-inoculated plants showed 13%, 13%, 11%, and 18% significantly (*P* < 0.05) higher root CuAO activity than non-AMF-inoculated plants at 0, 5, 10, and 15 days, respectively (Figure 7A). AMF-inoculated plants registered 15%, 29%, and 16% significantly (*P* < 0.05) higher root PAO activities over non-AMF-inoculated plants at 0, 5, and 10 days, respectively (Figure 7B).

### Changes in ROS and MDA Levels in Roots

**FIGURE 8:**
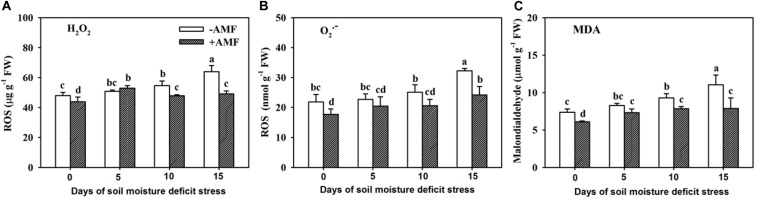
Contents of root hydrogen peroxide (H_2_O_2_) **(A)**, superoxide anion free radical (O_2_^–^) **(B)**, and malondialdehyde (MDA) **(C)** of trifoliate orange seedlings inoculated with and without *Funneliformis mosseae* during exposure to soil moisture deficit stress of 0-15 days. Data (mean ± SD, *n* = 4) followed by different letters above the bars indicate significant (*P* < 0.05) differences.

Root H_2_O_2_, O_2_^–^, and MDA concentrations in roots gradually increased with increasing span of SMDS, regardless of AMF- and non-AMF-colonized plants (Figures 8A–C). AMF-inoculated plants recorded significantly (*P* < 0.05) lower root H_2_O_2_, O_2_^–^, and MDA concentrations than non-AMF-inoculated plants at 0, 10, and 15 days, but not at 5 days, suggesting a lowering of H_2_O_2_, O_2_^–^, and MDA levels in AMF-inoculated trifoliate seedlings on exposure to SMDS.

## Discussion

In the present study, root AMF colonization gradually increased with the time of SMDS; however, it is difficult to distinguish whether the increase in mycorrhizal colonization is due to soil drought or contributed by the growth of mycorrhizal fungi. Augé (2001) summarized that SMDS affected root AMF colonization, increasing root colonization more often than decreasing it. An increase of root mycorrhizal colonization under sustained soil drought may be caused by the reduction in P diffusion rate in soil, which led to the decrease of P levels in plants, thereby stimulating the mycorrhizal fungal colonization (Augé, 2001). There was also an increase in Put content of roots during the SMDS process, indicating the relationship between mycorrhizal fungal colonization and root Put level. Earlier studies have shown the importance of PAs in root mycorrhizal colonization and arbuscule formation (Wu et al., 2012b; Salloum et al., 2018). Among PAs, Put, but not Spm and Spd, is the key factor involved in regulating the root mycorrhizal establishment (Wu et al., 2010).

In this study, AMF-inoculated plants showed higher total chlorophyll content than non-AMF-inoculated plants exposed to SMDS. PAs are bound to the pattern of thylakoid, prevented chlorophyll loss, not integrity, thus, stimulating the photosystem II in plants, which is an important mechanism operating in enhancing drought tolerance of plants (Unal et al., 2008; Munzi et al., 2009; Talaat and Shawky, 2013). PAs (e.g., Spm and Put) act as photo-protectors of PS II to exert a protective role in photo-adaptation under abiotic stress (Yaakoubi et al., 2014). However, leaf PA contents were not measured in this study, so it is difficult to establish the link between root PAs and total chlorophyll. Future work may focus on the relationship between mycorrhizal enhancement of total chlorophyll and regulation of PAs in leaves of host plants.

The present study also observed the reduction in leaf *Ψ* of both AMF- and non-AMF-inoculated seedlings under SMDS, indicating the effect of SMDS in limiting the water absorption of plants. On the other hand, during the SMDS, AMF-inoculated plants recorded significantly higher leaf *Ψ* than non-AMF-inoculated plants. In fact, mycorrhizal extraradical hyphae directly absorb water from the soil and transfer it back to arbuscule-containing cortical cells of roots for unloading to the host (Zhang et al., 2018). AMF-treated plants also showed higher root hydraulic conductivity than non-AMF-treated plants under soil drought conditions (Sánchez-Romera et al., 2016). PAs (e.g., Put) like compatible osmolytes prevented water loss of plants exposed to polyethylene glycol stress (Kotakis et al., 2014).

The synthetic precursors of PAs in plants mainly include L-Arg, L-Orn, Agm, and SAM (Liu et al., 2015). Simultaneously, L-Arg has an important role in sensing environmental changes and later aiding in adaptation to such abiotic stress (Yang and Gao, 2007). In plants, L-Arg is first transformed into Agm and then into Put, and L-Arg and L-Orn can be transformed into each other (Liu et al., 2015). In our study, we observed that AMF did not increase root L-Arg content, but SMDS of 5-10 days, to some extent, stimulated the concentration of four PA precursors in AM and non-AM trifoliate orange seedlings. Also, AMF inoculation substantially increased L-Orn, Agm, and SAM contents under SMDS, alongside higher PA synthase (e.g., ADC, ODC, SPDS, and SPMS) activity. These observations meant mycorrhizal fungi may accelerate the conversion of L-Arg into Agm by ADC, thereby resulting in an increased concentration of Agm without obvious changes in L-Arg. At the same time, AMF also accelerated the accumulation of other PA precursors (L-Orn, Agm, and SAM), later transformed into Put and Spd by combined effect of ODC, SPDS, and SPMS (Kusano et al., 2008; Vera-Sirera et al., 2010; Liu et al., 2015). Such changes in AMF-inoculated plants exposed to SMDS could be considered as SMDS responsive mechanism, beneficial for AMF to regulate PA homeostasis in roots, so that AMF-inoculated plants develop an elevated ability to tolerate SMDS than non-AMF-inoculated plants.

In our study, we analyzed the changes in concentration of diamine (Put and Cad), triamine (Spd), and tetramine (Spm) in roots in response to SMDS and AMF inoculation. The changed pattern of root Spd and Spm concentrations in AMF- and non-AMF-inoculated plants was different in response to SMDS. We further observed two contrasting trends in Put and Cad concentration between AMF- and non-AMF-inoculated plants. The results suggested that diamine of AMF-treated plants was not activated up to 10 days of SMDS, while non-AMF-treated plants experienced a reverse change, indicating that AMF-treated plants exhibited a relatively higher tolerance against SMDS than non-AMF-treated plants. Interestingly, AMF-treated plants possessed higher diamine content at the beginning of exposure to SMDS. AMF-colonized plants showed higher root Put and Cad content than non-AMF-colonized plants at 0, 5, and 15 days of SMDS. Wu et al. (2012a) earlier reported an increase in root Put level in red tangerine after inoculation with *F*. *mosseae* under ample water conditions. Zhang et al. (2020) also noted higher root Put and Cad concentration in trifoliate orange colonized by *F*. *mosseae* under both WW and drought stress conditions. Put is associated with stress tolerance by abscisic acid regulation and antioxidant system activation (Pál et al., 2015). It is concluded that higher diamine content in AM versus non-AM plants is considered beneficial to enhance the SMDS tolerance of AM plants. On the other hand, Spd, a downstream product of Put (Liu et al., 2015), was relatively lower in AMF- than in non-AMF-colonized trifoliate orange during SMDS. Root Spm was lower in AM plants than in non-AM plants in the beginning of the stress, but ended up with higher Spm. Higher Spm in AMF versus non-AMF plants under SMDS may increase stress-related gene expression level to protect against any potential stress damage (Fu et al., 2014; Pál et al., 2015). Roots are the main site for synthesis of Put (Moschou et al., 2008). Mycorrhizal fungi colonize the root system and, thus, stimulate the content of root Put and Cad, while Spd and Spm are downstream products of Put and synthesized mainly in shoots (Moschou et al., 2008), thus, exhibiting the diminishing response in AMF-inoculated plants.

Past studies have confirmed that AMF colonization alleviated the oxidative burst of host plants induced by drought stress, thus, maintaining low ROS levels (Gholinezhad et al., 2020; Langeroodi et al., 2020; Zou et al., 2020). In our study, we observed relatively higher CuAO and PAO activity in AMF-inoculated plants than non-AMF-inoculated plants under SMDS, which is in agreement with previous studies under varying stress conditions (Xue et al., 2009; Toumi et al., 2010; Zhang et al., 2020). Normally, drought stress causes PA catabolism to result in ROS (e.g., H_2_O_2_) burst (Gupta et al., 2013). However, our study still showed relatively lower root ROS (e.g., H_2_O_2_ and O_2_^–^) levels and a low degree of root membrane lipid peroxidation (e.g., MDA) in AMF-inoculated trifoliate orange seedlings than in non-AMF-inoculated seedlings exposed to SMDS. He et al. (2020) earlier observed similar events in trifoliate orange inoculated with *F*. *mosseae* under both WW and drought stress. In fact, inoculation with AMF activated the antioxidant defense system of both host plants and AMF to respond to drought-induced ROS burst (Zou et al., 2020). In our other study, colonization by *F*. *mosseae* significantly upregulated the expression levels of *PtMn-SOD*, *PtCu/Zn-SOD*, and *PtCAT1* genes in the roots of trifoliate orange exposed to a 7-week SMDS (50% maximum water holding capacity) (Zhang et al., 2020). In addition, PA application is involved in the elicitation of antioxidant enzyme defense systems in stressed plants (Wu et al., 2020). It seems that PA-induced H_2_O_2_ could trigger the antioxidant defense system of plants, thereby bringing PAs into operation as scavengers of free radicals (Gupta et al., 2013). More in-depth studies are needed to elucidate the signal role of H_2_O_2_ induced by PA catabolism in response to SMDS under mycorrhization.

## Conclusion

In this study, AMF facilitated the accumulation of Put, Cad, and PA precursors and increased PA synthase activity, accompanied with low oxidative burst in trifoliate orange in response to SMDS. Thus, AMF modulated the PA homeostasis of host plants to effectively cope with SMDS. Such results provide strong clues in understanding the physiological mechanisms imparting an enhanced SMDS tolerance in AMF-inoculated plants. Future studies should highlight the mechanisms regarding AMF-induced PA regulatory network at the molecular level and the involvement of associated signals.

## Data Availability Statement

The original contributions presented in the study are included in the article/supplementary material, further inquiries can be directed to the corresponding author/s.

## Author Contributions

FZ and Q-SW conceived and designed the experiments. FZ and Y-NZ performed the experiments. Y-NZ, FZ, AKS, Q-SW, and KK analyzed the data. FZ and Q-SW prepared the figures. Y-NZ wrote the manuscript. Y-NZ, AKS, Q-SW, and KK revised the manuscript. All authors contributed to the article and approved the submitted version.

## Conflict of Interest

The authors declare that the research was conducted in the absence of any commercial or financial relationships that could be construed as a potential conflict of interest.
